# The Scope of Big Data in One Medicine: Unprecedented Opportunities and Challenges

**DOI:** 10.3389/fvets.2017.00194

**Published:** 2017-11-16

**Authors:** Molly E. McCue, Annette M. McCoy

**Affiliations:** ^1^Equine Genetics and Genomics Laboratory, Veterinary Population Medicine, University of Minnesota, St Paul, MN, United States; ^2^Veterinary Clinical Medicine, University of Illinois Urbana-Champaign, Urbana, IL, United States

**Keywords:** deep phenotyping, multilayer disease module, network medicine, bioinformatics, structural informatics, clinical informatics, genetic epidemiology, environmental epidemiology

## Abstract

Advances in high-throughput molecular biology and electronic health records (EHR), coupled with increasing computer capabilities have resulted in an increased interest in the use of big data in health care. Big data require collection and analysis of data at an unprecedented scale and represents a paradigm shift in health care, offering (1) the capacity to generate new knowledge more quickly than traditional scientific approaches; (2) unbiased collection and analysis of data; and (3) a holistic understanding of biology and pathophysiology. Big data promises more *personalized* and *precision* medicine for patients with improved accuracy and earlier diagnosis, and therapy tailored to an individual’s unique combination of genes, environmental risk, and precise disease phenotype. This promise comes from data collected from numerous sources, ranging from molecules to cells, to tissues, to individuals and populations—and the integration of these data into networks that improve understanding of heath and disease. Big data-driven science should play a role in propelling comparative medicine and “one medicine” (i.e., the shared physiology, pathophysiology, and disease risk factors across species) forward. Merging of data from EHR across institutions will give access to patient data on a scale previously unimaginable, allowing for precise phenotype definition and objective evaluation of risk factors and response to therapy. High-throughput molecular data will give insight into previously unexplored molecular pathophysiology and disease etiology. Investigation and integration of big data from a variety of sources will result in stronger parallels drawn at the molecular level between human and animal disease, allow for predictive modeling of infectious disease and identification of key areas of intervention, and facilitate step-changes in our understanding of disease that can make a substantial impact on animal and human health. However, the use of big data comes with significant challenges. Here we explore the scope of “big data,” including its opportunities, its limitations, and what is needed capitalize on big data in one medicine.

## Overview and Introduction

“Big data” has become a catch phrase across many industries including medicine. As of this writing, a PubMed search for the term “big data” retrieves 10,015 entries, each detailing some aspect of big data in human or veterinary medicine, public heath, veterinary epidemiology, environmental or ecosystem health, and animal husbandry, among others. Occasionally, these papers encompass big data at the intersection of one or more of the above fields, but they generally deal with only one aspect or application of big data. Even for those investigators working with and applying big data to understand health and disease, an individual’s definition of big data is often limited to its use within a particular field of study. Therefore, the goals of this review are to demonstrate the scope of big data in medicine, broaden the reader’s perspective regarding the opportunities for integration of big data across different disciplines, and show how big data can be applied to “one medicine.”

Before delving into the breadth and depth of big data, we start by introducing the reader to our definitions of “one medicine” and “big data” as they will be used throughout this review.

## One Medicine

Similar to big data, the terms “one medicine” and “one health” are popular catch phrases, with definitions that vary depending on the source. For the purposes of this review, we use a definition of “one medicine” similar to that first proposed by Schwabe in 1984 and extended by Zinsstag in 2011 (1). This definition acknowledges the body of knowledge, including physiology, pathology, and anatomy, which is shared across species, and further, that disease processes are defined and impacted by processes at the molecular, cellular, tissue, whole organism, and population levels. This definition also acknowledges that disease processes are impacted by intrinsic (age, gender, behaviors, comorbidities, etc.) and extrinsic (environmental) factors. Our definition of “one medicine” also has a clinical connotation—is focused toward understanding disease and disease processes; although it does not exclude identifying disease risk factors or disease prevention. We use “one medicine” as the *union* of the overlapping disciplines of human and veterinary medicine and shared environmental risks. This is in sharp contrast to the definition of “one medicine,” and more recently “one health,” that has been largely developed in the public health community. This definition was focused initially on the contribution of veterinary medicine to public health and has more recently extended the study of the environment to include ecosystem health. The use of this definition of “one health” tends to be focused on the *intersection* between human health, animal health, and the environment/ecosystem (1).

## What is Big Data?

“Big” is a relative term when it comes to data (2). One practical definition of “big data” is “datasets so large or complex that traditional data processing methods are inadequate” (3). While this definition captures the reality of big data, the definition originally proposed by Gartner, which describes big data by its volume, variety, and velocity (the 3V’s), has been adopted by many authors (4). Additional V’s utilized by others to describe big data include veracity, value, and variability (Table 1) (5). Regardless of the exact definition, the volume of “big” datasets, along with their complexity and diversity, requires unique data storage and retrieval solutions, and makes these data difficult to manipulate and analyze. Several recent reviews discuss computational and storage solutions, such as parallel computing with Hadoop and cloud computing. The reader is referred to these papers for an in depth discussion of these resources (6–8).

**Table 1:** The Vs of big data.

Volume	High-throughput technologies for gathering data and/or continuous gathering of clinical population data
Variety	Heterogeneous structured and unstructured data from various sources both qualitative (text mining medical records) and quantitative (medical images, high-throughput omics data, clinical laboratory tests, environmental data from sensors, etc.)
Velocity	High-speed processing for fast decision-making in real time or near real time
Variability	Consistency or inconsistency of the data over time
Veracity	Data with variable quality and data from uncontrolled environments
Value	Data relevant to the health of individuals or populations and data from longitudinal studies

*Big data is defined in terms of either the first 3, or all 6, Vs*.

### Volume—How Big Is “Big Data”?

The volumes of available datasets are growing exponentially, with modern studies yielding terabytes, petabytes, or exabytes of data (2). In an effort to develop “a precise, well-formed, and unambiguous” definition of big data in health care, Baro et al. conducted a query of the literature for the term “big data” and identified 196 papers directly related to human health care. Of these, 48 included datasets and were mined for the number of statistical individuals (*n*, which may be greater than the number of physical individuals), and the number of variables (*p*) measured for each individual (e.g., clinical data, ‘omics data) (9). Based on this review, these authors proposed a quantitative cut-off for big data based solely on the total number of data points within the dataset, defining “big datasets” as those in which log (*n* × *p*) ≥ 7 (9).

These authors note that health-care big data can be classified into three categories based on the number of individuals and the number of variables. The first category, typical of big data in ‘omics studies, is characterized by massive numbers of data points (100 s to millions), collected on a limited number (100–1,000 s) of individuals; i.e., small *n*, high *p*. The second category, which encompasses medical or biomedical informatics studies, is typified by a moderate to large number of individuals in which a moderate to large number of variables are measured; i.e., high *n*, high *p*. The third category includes public health big data and is characterized by a large number of individuals with a limited number of measured variables; i.e., high *n*, small *p*. It is important to note that the definition of big data proposed by Baro et al. uses a different unit of measurement than the quantitative definitions of other authors, who describe big data using the size of the resulting data, and quantify big data as terabytes or larger. Using a volume-based definition, most epidemiologic datasets “barely pass the big data threshold” in volume (2). However, even marginally large epidemiologic datasets still have other important characteristics of big data such as velocity, variety, variability, veracity, and value (Table 1). Thus, Baro et al. present an attractive definition of big data in health care, as their definition of big data captures not only the size of these datasets but also captures the breadth and/or complexity of the data (9).

### Variety—Where Does “Big Data” Come From?

**Figure 1:**
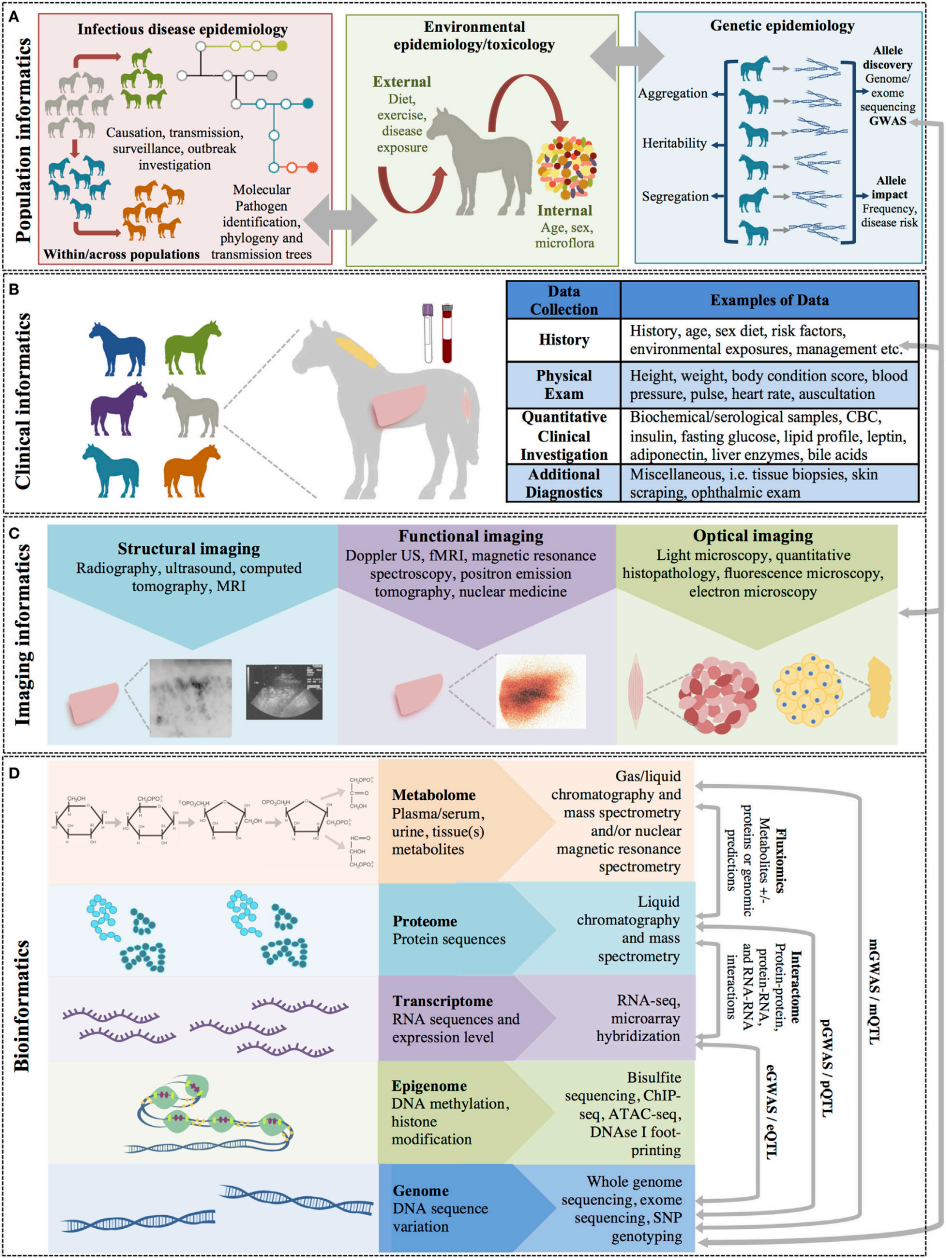
The multiple levels of biomedical informatics data. **(A)** Population health informatics focuses on the study of infectious and genetic disease in populations and the impacts of environmental exposures (i.e., the exposome: internal, general external, and specific external environments). Although metagenomics is the study of the small molecules of the genome of microorganiosms, the microbiome is considered an environmental factor by many investigators. **(B)** Clinical informatics includes all quantitative and qualitative clinical measures made on patients including history, physical examinations, clinical laboratory testing, and other clinical diagnostic procedures. **(C)** Imaging informatics encompasses measures made at the tissue or organ level and includes structural and functional imaging studies as well as histopathology and other microscopic studies. **(D)** Bioinformatics encompasses the largest level and includes all measurements of small molecules (i.e., the ‘omics studies). The bioinformatics level also incorporates studies of the interactions between molecules of the same of different molecular levels within a cell (the “interactome”) and describes the molecular phenotype of health and disease.

**Figure 2:**
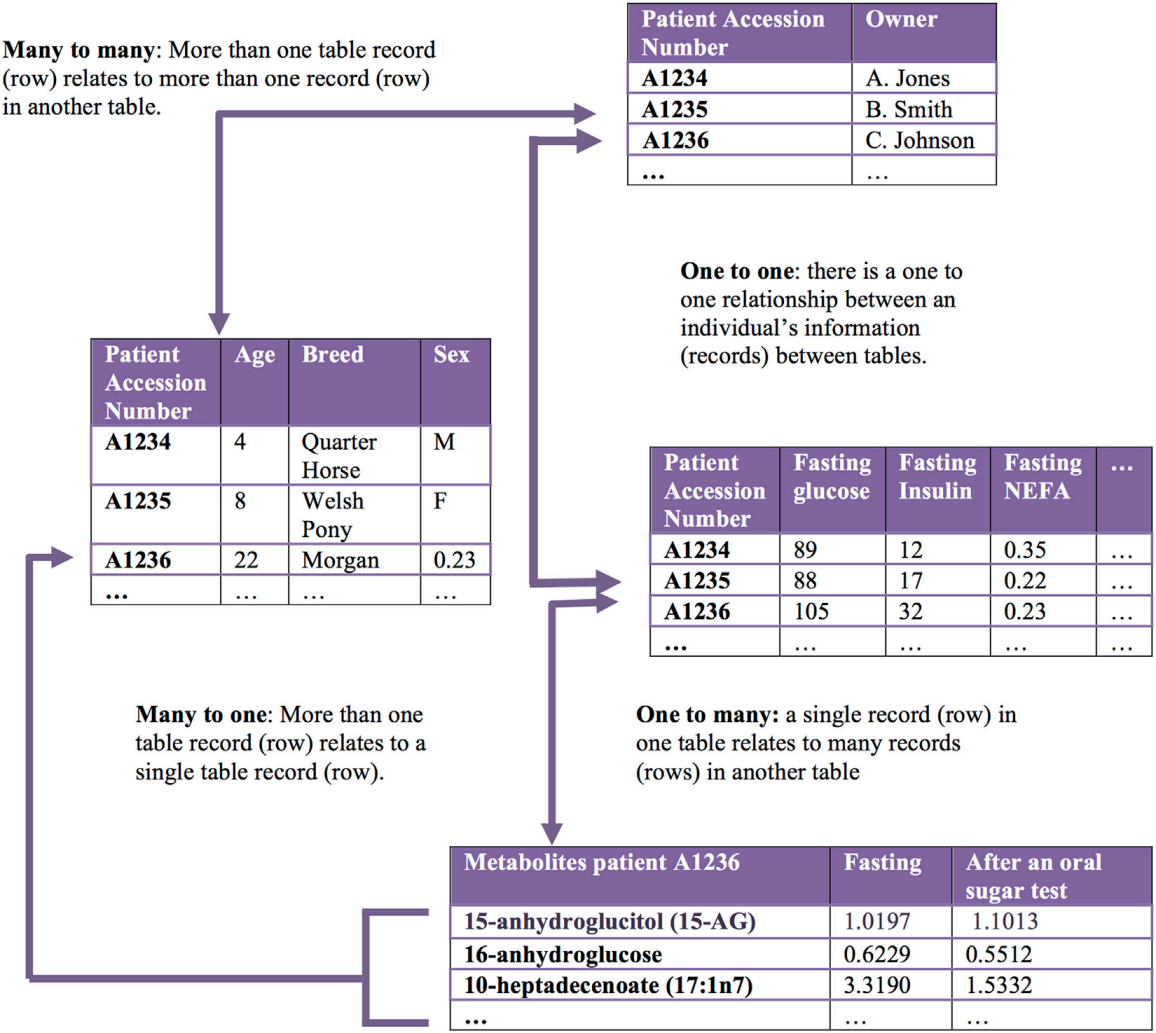
Relational databases capture related datasets. A relational database organizes a collection of multiple related datasets. Each dataset is organized within a table by rows and columns. Each table relates to one or more tables in the relational database, and tables communicate with each other to share information. Each table is a “relation,” which contains one or more data columns. Each row in a table is considered a “record” and contains unique data in the corresponding columns. One or more record(s) has data within column(s) that relate to one or many records contained in other tables.

Big data in health care comes from numerous different sources across many levels, from molecular to cellular, whole organ, individual (i.e., “clinical” measurements), environmental, and population levels, with a variety of different possible measurements made at each of these levels (Figure 1). The data gathered from these sources may be structured or unstructured. Structured data have a high degree of organization, which makes it amenable to representation within the rows and columns of a data matrix. Structured data are often stored in relational databases (Figure 2). Once structured data are defined in terms of rows and columns within a database, it is simple to enter, store, query with various search algorithms, and analyze using computers (4, 10). Examples of structured data in health care include high-throughput ‘omics data, clinical laboratory tests, and environmental data from sensors. In contrast to structured data, unstructured data has no predefined organization, and while it may have its own internal structure, it does not conform to rows and columns and is not easily stored in databases (4, 10). Unstructured data is meant for processing by the human brain and comes from various sources including text (health record written notes, manuscripts, laboratory reports) and medical imaging [magnetic resonance imaging (MRI), radiographs, computed tomography (CT)]. While these data can be coded for capture in a structured format, some information is almost inevitably lost in this process. It has been estimated that approximately 80% of information in the human health-care industry is unstructured (11, 12). The heterogeneous nature of these data makes aggregation and interpretation difficult.

### Velocity—How Fast Does “Big Data” Accumulate?

Velocity refers to how frequently the data updates, or to the data’s growth over time (13). Data that updates in real time, or near real time, has added value as it can help researchers and clinicians make decisions that provide strategic advantages; for example, in modeling, the specific impact of preventative, treatment, or management decisions. Data velocity is a particularly important feature of population- or public health-based datasets as receiving and analyzing data in near real time can improve understanding of disease spread and outcomes in outbreaks.

### Variability—How Does “Big Data” Change?

Variability in big data refers to the data’s completeness and how the data may, or may not, change over time. These characteristics pose challenges for many statistical analyses and data modeling techniques and require special consideration in data quality control, including the decision to impute missing data values, and how to handle repeated data measures (3, 14, 15). Variability also captures the complexity of biomedical data, even when the data comes from a single source. For example, gene expression data can be different in different tissues and changes dynamically over time during development and in response to different environmental stimuli. Each gene can express a variety of transcripts with differing effects. Further, transcripts from a single gene locus can have differing effects in different tissues or at different times in development (pleiotropy). This aspect of big data makes interpretation within the correct context particularly crucial and may affect the ability to extrapolate findings into a novel context (14).

### Veracity—How Much of “Big Data” Can We Believe?

Datasets may vary with respect to noise, redundancy, and consistency, leading to uncertainty in data quality and making the data difficult to validate. Veracity defines the accuracy and truthfulness of a dataset. Controlling for veracity in a dataset requires careful data “cleaning” and rigorous integrity checks before performing additional data analysis (15).

### Value—Why Should We Care about “Big Data”?

Despite many of the challenges outlined above, the value of big data lies in the potential to gain insight into complex conditions affecting the health of individuals and populations that have historically been resistant to robust analysis. Importantly, big data has the potential to greatly expand knowledge for many clinical conditions in which collecting prospective, structured data is time- and cost-prohibitive (16). It is important to recognize that agreement within biomedical research communities regarding best practices for data collection, storage, quality control, and analysis can enhance the value of big datasets. Standardized methodologies allow data to be used repeatedly to answer different questions, as well as more direct comparisons to be made between outcomes in different patient cohorts (15).

## Opportunities for Big Data in One Medicine

Over the course of history, medicine has been considered as both an art and a science. Traditionally, health-care professionals largely used experience and observation to guide diagnoses and treatment decisions. However, in recent decades, advances in high-throughput molecular biology and electronic health records (EHRs), coupled with increasing computer capabilities from terabytes to petabytes, have thrust data-driven medicine to the forefront (14, 17). There is now general acceptance that evidence-based medicine—and even more recently, truly *personalized* and *precision* medicine—should constitute the gold standard of care (18, 19). Yet, this new standard requires collection and analysis of data at an unprecedented scale—in other words, “big data.” This represents a paradigm shift in health care, but one with the potential for a huge pay-off in terms of understanding disease pathophysiology and improved patient outcomes. In fact, the use of big data in translational research has several advantages that complement traditional, theory-driven, direct experimentation:

1. Big data approaches have *the capacity to generate new knowledge more quickly* than the traditional paradigm of scientific discovery (5, 12, 16, 20). For example, modern high-throughput technologies can generate thousands to millions of data points from thousands of individuals, within a matter of hours to days. Rigorous analysis of this data leads to new information about the system, eventually adding to our knowledge of health and disease.
2. Big data approaches are often *unbiased* by prior knowledge. Unbiased collection and analysis of data, and discovery of important patterns, supports evidence-based medicine by constructing more relevant predictive models allowing more accurate assessment of disease risk and reoccurrence, as well as improved estimations of prognosis (21, 22). Further, analysis of relevant data can be used to formulate specific testable hypotheses about biologic systems.
3. Big data approaches are *holistic*. Big data is not limited to a single pathway, cell, tissue, individual, or population—it considers disease across molecules, cells, tissues, individuals, populations, and environmental exposures. This holistic approach better captures the biology or pathophysiology of interest (20, 23).

As a result of these benefits, it is easy to envision how big data will result in more tailored health care. Better recapitulation of pathophysiology leads to an improved understanding of disease etiology and progression, resulting in improved accuracy and earlier diagnosis, and application of therapy personalized to an individual’s unique combination of genes, environmental risk, and precise disease phenotype. Faster identification of high-risk patients results in more timely treatment decisions. Amalgamation of big data across institutions can identify rare diseases and rare drug or reactions or interactions (23, 24).

The potential rewards of big data-driven scientific discovery have translated into enthusiasm and application of big data approaches in human health care, and its use is driving scientific breakthroughs. Arguably, for veterinary and comparative medicine, big data-driven science could play an even bigger role in moving these fields forward. The breadth of data now available from the EHR and the merging of this data across institutions, will give health-care researchers access to patient data on a scale previously unimaginable (25, 26). This is particularly true for veterinary and translational researchers who often struggle to compile large enough cohorts of patients from a single institution/practice to make meaningful statistical comparisons and draw generalizable conclusions. Collection of high-throughput molecular data will provide an understanding of molecular pathophysiology and disease etiology previously unexplored in veterinary patients. More importantly, these data will also push comparative medicine in to new territory, where parallels between human and animal disease will be drawn at the molecular level, and similarities and differences will create knowledge across species.

Big data will never completely replace traditional, theory-driven scientific discovery; these approaches will always be important for validation of biological mechanisms. However, it can facilitate step-changes in our understanding of disease that can make a substantial impact on clinical practice in both human and veterinary medicine. However, the big data revolution is still in its infancy, with significant challenges to overcome before fully claiming its benefits (12). Here, we explore the scope of “big data” in health care, its opportunities, its limitations, and what is needed capitalize on big data in one medicine.

## Levels of Biomedical Informatics Data

Biomedical or health-care informatics is an interdisciplinary field that uses biomedical data obtained from numerous sources, ranging from molecules to individuals and populations, to improve health through scientific inquiry and problem solving (modeling, simulation, and experimentation) and to improve clinical and population-level decision-making (translation). Biomedical informatics can be broken down into subfields that use different types (or levels) of data to understand disease within individuals and populations (Figure 1).

### Bioinformatics

Bioinformatics is defined as the study of complex biological data arising from molecules and cellular processes and aims to characterize and quantify the interactions of biological molecules that translate into the structure, function, and dynamics of an individual (11). This field has grown exponentially in recent years due to the development of high-throughput technologies such as next-generation sequencing (NGS) and quantitative mass spectrometry, which can capture massive amounts of data from an individual. Molecular level data, including genes, transcripts, proteins, and metabolites, can be collected from different tissues, single cells, or across different conditions (e.g., before and after disease, before and after treatment, at different time points in development) to provide cell and/or context-specific insights and to understand the interactome, or the entirety of molecular interactions within a cell. ‘Omics data, or ‘omics profiling, refers to the collection of these high-throughput molecular data sets. Bioinformatics leverages ‘omics data to interrogate biologic function and dysfunction and to understand how changes at the molecular level translate to disease states by relating the ‘omics profile of each individual, (i.e., the genome, transcriptome, proteome, metabolome, etc.) to the phenotypes obtained from clinical observations, medical images, and other physiological data (5).

#### Genomics

Genomics aims to characterize the sequence, structure, function, and evolution of genomes, or the entirety of an individual or species’ genetic material. Genes and genetic alleles are the static upstream “blueprint” controlling dynamic biological processes. Differences between individual’s genomes are due to a variety of different genetic alleles including single base-pair changes [single-nucleotide polymorphisms (SNPs)], insertions or deletions of one to millions of base pairs, copy number variants (duplications, deletions, etc., CNVs) and genetic inversions. Genetic alleles exert their influence by altering gene expression, gene regulatory mechanisms [transcription factors, microRNA (miRNA), etc.], or proteins (abundance, function, or regulation), which in turn alter the structure and/or function of cells and tissues (e.g., metabolic pathway activity and metabolite abundance and/or ratios) (Figure 1D). In this way, genetic alterations are reflected at several molecular levels as molecular traits (Figure 1D), which are precursors for the “endpoint of interest” such as disease or a clinical diagnostic measurement (27). Variation in individual genomes can be captured by SNP or CNV genotyping arrays and by sequencing of specific regions or genes using Sanger or NGS [i.e., whole exome sequencing and targeted or untargeted whole genome sequencing (WGS)]. NGS methods, in particular, whole-genome sequencing, have become commonplace in human and veterinary medicine due to the reduction in sequencing costs. In the last 15 years, the cost of sequencing an entire mammalian genome has decreased by a factor of ~1 million, and individual genomes can now be sequenced for as little as $1,000, depending on the desired depth of sequence coverage (28).

#### Epigenomics

Epi literally means “on top of”; thus, epigenomics signifies processes that are happening “on top of” the genome. Epigenetic modifications are heritable, but reversible modifications of a cell or organism’s DNA, histones, and/or chromatin structure that affect gene expression without altering the underlying DNA sequence. Epigenetic modifications play an important role in gene expression and regulation, are involved in numerous cellular processes such as cell differentiation and development, and are important for phenotypic plasticity (i.e., phenotypic change in response to environmental change) (29). Epigenomics is concerned with understanding the landscape of methylation, histone modification, and chromatin structure on a global level and these how changes impact transcriptional regulation, cellular differentiation, and cellular phenotypes. This landscape can be defined by several NGS technologies, including chromatin immunoprecipitation (ChIP)-Seq and bisulfite sequencing. ChIP-Seq combines ChIP, which pulls down DNA bound to a protein of interest (e.g., important histone modification markers such as histone H3 lysine 4 trimethylation), and sequencing to localize DNA binding sites and define chromatin architecture and accessibility in a genomic region of interest (30). Open chromatin (which is transcriptionally active) can also be identified on a genome-wide level through other methods including transposase-accessible chromatin sequencing (ATAC-seq) and DNAse I footprinting (31, 32). In contrast, bisulfite sequencing detects another mechanism for transcriptional regulation in mammalian DNA, the addition of a methyl group to CpG dinucleotides. Bisulfite sequencing has resolution to the base-pair level and can provide insight into key processes such as genomic imprinting and X-chromosome inactivation (33).

#### Transcriptomics

The transcriptome is the sum total of all the ribonucleic acid (RNA) molecules [i.e., messenger RNA (mRNA), miRNA, non-coding RNA (ncRNA), etc.] expressed in a cell, tissue, or organism. Altered expression and regulation of genes is reflected in tissue transcriptomes. Genes can be differentially expressed between tissues, physiologic or disease states, or developmental time-points. Differential expression can also be defined by the expression of alternate transcripts that affect function differently. Quantifying the transcriptomes of different cells and tissues across individuals and different states can lead to insight into differing biologic function between states, and gene co-expression can give insight into shared regulation between genes. Microarray hybridization or RNA sequencing using next-generation technologies (RNA-seq) are used for comprehensive quantitation of gene expression in cells or tissues (34, 35).

#### Understanding Genome Function through Genomic, Epigenomic, and Transcriptomic Data Integration

The regulation of gene expression and protein function are key factors resulting in cellular differentiation and function. Alterations at the gene or protein level may lead to cell- and organism-level genotypes of interest. Many recent advances in biology have been driven by genome sequence information. However, gene expression within a given cell is affected at several levels, including (1) epigenetic modification and genomic variation impacting transcription factor binding; (2) RNA transcription, processing, and transportation; (3) protein translation; and (4) posttranslational protein processing and degradation. Further, regulatory proteins that bind to DNA and RNA play an important role by positively or negatively regulating specific protein level and function in a cell. Understanding the complexity of these genetic and epigenetic interactions is a key component to understanding how changes in the genome can predict complex phenotypes, including those resulting in disease.

In humans, the Encyclopedia of DNA Elements (ENCODE) Consortium and epigenome consortia such as the Blueprint Epigenome Consortium are working to build a comprehensive list of functional elements in the human genome, including those that act at the epigenetic, genomic, RNA, and protein levels (29, 36, 37). These consortia have a goal of understanding the regulatory elements that control all genes in cells under all circumstances where that particular gene is active. Expansion of these efforts into model organisms such as *Mus musculus* and *Drosophila* have shown that transcriptome complexity and gene expression differs significantly between species; for example, although a subset of core regulatory programs is conserved, nearly 50% of these elements differ between mice and humans (38). These data highlight the need to perform genome-wide identification of functional elements in multiple species of veterinary interest to facilitate the dissection of genotype-to-phenotype relationships. With the recent advances in NGS technology, WGS, transcriptome sequencing, and quantification, and methods to identify epigenetic modifications at the genome level can be performed without the development of species-specific tools, thus enabling ENCODE and Blueprint consortia-like efforts in domestic animals for the first time. A coordinated international effort, the Functional Annotation of Animal Genomes project (FAANG) was initiated in 2014 to accelerate genome-to-phenome identification in several animal species of veterinary interest including the cow, pig, horse, chicken, sheep, and goat (39). In the first phases of this effort, a number of investigations have been proposed across 80–105 tissues, depending on the species (39). These include: (1) WGS; (2) whole genome bisulfite sequencing; (3) RNA sequencing (mRNA, miRNA, ncRNA) and transcriptome assembly; (4) ATAC-seq; (5) ChIP-seq with DNAse I; (6) histone modification marks, insulator-binding protein CCCTC-binding factor, and important transcription factors; and (7) study of the genome-wide chromatin interactome using Hi-C. Work is ongoing among members of the FAANG project to standardize collection techniques, experimental protocols, and data analysis pipelines to maximize the utility of the data produced by this effort.

#### Proteomics

Proteomics is the large-scale study of proteins in the proteome; that is, the entire complement of proteins that is expressed by a cell, tissue, or organism, or in a particular biologic context. Altered protein abundances are reflected in the proteome. Similar to the transcriptome, the proteome is not constant; it differs from cell-to-cell, tissue-to-tissue, and between individuals; it also changes over time. The proteome somewhat reflects the underlying transcriptome; however, protein activity is also modulated by many additional factors. The goal of proteomics is to obtain a more global and integrated view of biology by considering all the proteins of a cell/tissue rather than studying each protein individually. Questions about proteins that can be addressed by this approach include (1) when and where are proteins expressed; (2) what is the steady-state abundance of proteins and the rate of protein production and degradation; (3) how are proteins modified (e.g., alternative splicing, posttranslational modifications); (4) how and where do proteins move within a cell; (5) how do proteins function within metabolic pathways; and (6) what are the significant protein–protein interactions within a cell, tissue, physiologic/pathologic state, etc. (40).

Several high-throughput technologies that generate large amounts of data have been developed to investigate the proteome. Mass-spectrometry (MS), particularly tandem mass spectrometry (MS/MS), is frequently utilized in discovery (shotgun) proteomics to determine the relative abundances of peptides. Recently, chemical labeling techniques, such as isobaric tags for relative and absolute quantification (iTRAQ), have further improved quantification accuracy (41). Similarly, recent advances including the development of specific affinity chromatography reagents have allows for the enrichment of phosphorylated peptides, which enables robust phosphoproteomics (42). Proteomics coupled with metabolomics (below) has also led to major advances in the understanding of enzyme kinetics *in vivo*, as the rate of an enzymatic reaction can be estimated by dividing the metabolite flux through the enzyme by the enzyme abundance (as determined by quantitative proteomics) in the system (43).

#### Metabolomics

The metabolome is the set of all small molecules present within a tissue, system, or organism including nucleotides, amino acids, carbohydrates, sugars, sugar phosphates, nucleotides, bile acids, sterols, carboxylic acids, phospholipids, triglycerides, and fatty acids, among others. Metabolomics is the study of cellular processes *via* quantification of these small molecules or metabolites. Specific quantification of lipids and related molecules (fatty acyls, glycerolipids, glycerophospholipids, sphingolipids, saccharolipids, and polyketides) has developed as an important sub-field of metabolomics referred to as *lipidomics* (44). The effects of altered gene expression, gene regulatory mechanisms, protein abundance, protein function, and protein regulation, as well as environmental factors, including changes in the microbiome, are reflected in the metabolome (Figure 1). Methods to determine metabolite levels can be divided into “targeted” methods designed for routine quantification of a specific set of pre-defined metabolites (typically <200), and “non-targeted” methods that can potentially quantify thousands of metabolites not selected in advance (45). The latter data-driven, global discovery methods are useful for identifying novel targets, but often require more focused follow-up with a targeted approach to facilitate biologic interpretation. In both targeted and untargeted metabolomics, metabolites are measured either by mass spectrometry (MS) in combination with liquid and/or gas chromatography (46) or by nuclear magnetic resonance spectroscopy (MRS) (47).

Because many diseases, particularly chronic complex diseases, are caused by altered metabolism, metabolomics approaches are increasingly being used in medicine. While metabolites can be quantified in individual tissues, measuring the plasma, serum, or urine metabolomes provides a footprint of the whole body’s metabolic processes (48) and have the advantage of being obtained through minimally invasive means. Further, because the serum/plasma metabolome represents the summation of the metabolic processes across all tissues relevant to metabolism, evaluation of metabolite abundance or the ratios between pairs of metabolites provides information about disruption in metabolic processes by both endogenous and exogenous pathways (e.g., xenobiotics, gut microbiome metabolites, environmental pollutants).

Metabolomics provides information on the metabolites in a biological sample, but this is only a snapshot of a dynamic process. *Fluxomics* extends metabolomics a step further by attempting to identify or predict the rates of metabolic reactions in an individual or tissue. The metabolic flux is typically measured either with flux balance analysis, which estimates flux using stoichiometric constraints or ^13^C-fluxomics, in which metabolic precursors enriched with ^13^C are introduced into the system (49, 50). Flux predictions can be improved by coupling with proteomic analysis to quantify the total amount of a given enzyme. Although it cannot be measured directly, metabolic flux is a critical link between genes, proteins, and phenotype.

#### Metagenomics

Metagenomics is broadly defined as the study of genetic material recovered directly from environmental samples, including bacteria, fungi, viruses, and other microbes. In health care, the field of metagenomics has mostly been restricted to the study of the microbiome (the community of commensal, symbiotic, and pathogenic microorganisms living on/in an individual), in particular, the gut microbiome (51). 16S RNA sequencing and, more recently, shotgun NGS has been used to identify the number and diversity of species living within the human gastrointestinal tract. This approach has led to a growing body of evidence supporting the symbiotic relationship between the intestinal microbiome and host metabolic homeostasis, with dysbiosis being implicated in various disease processes and pathological states (52–55). There is much interest in studying the microbiome in health and disease states as it has the potential to identify opportunities for prevention or therapeutic intervention with prebiotics, probiotics, and/or antibiotics. In fact, investigation of the microbiome is perhaps one of largest “big data” research areas in medicine today. A PubMed search using the terms microbiota, microbiome AND human returns 15,776 articles published since 1958, of which 12,282 (78%) have been published in the past 5 years. The body of veterinary literature is smaller (1,772 articles since 1988), but it is also rapidly growing, with 85% of these articles published since 2012.

Metagenomics allows researchers to access genetic diversity of microbial communities, and thereby infer metabolic diversity, but it cannot show which of these metabolic processes are active. The extraction and analysis of mRNA from the microbiome provides information on the expression profiles the constituent microbes (i.e., the metatranscriptome). *Metatranscriptomics* work is still in its infancy, due to technical difficulties including the short half-life of mRNA.

## Imaging Informatics

Imagining informatics is concerned with capturing data at the tissue level, including both anatomical (structural) information and, in some circumstances, functional information (Figure 1C). Imaging plays a central role in disease diagnosis in both human and veterinary medicine, and advances in imaging techniques over the last few decades have had a large impact on diagnostic capabilities by both increasing the detail in which the body can be imaged, and by adding functional information, particularly for the cardiovascular system (Doppler ultrasound [U/S]) and the brain [functional magnetic resonance imaging (fMRI)] (5, 56).

Structural imaging is focused on high anatomical-spatial resolution including the clear depiction of abnormalities. Structural imaging modalities include radiography, ultrasound (U/S), CT, and MRI. Optical imaging modalities include light microscopy (e.g., quantitative histopathology), fluorescence microscopy (e.g., confocal microscopy), and electron microscopy. Some structural imaging modalities are organ-specific, such as those developed for ophthalmologic imaging including retinal photography, auto-fluorescence, fluorescein angiography, and optical coherence tomography (56). Functional imaging modalities infer function by capturing structural changes over time; these modalities include MRS, positron emission tomography, and nuclear medicine imaging, in addition to Doppler U/S and fMRI.

Regardless of the modality, biomedical informatics has shifted toward digital capture of structural and functional images. A major goal of the methods of imaging informatics is to extract information about anatomy and to collect features that will be useful for characterizing abnormalities based on morphologic changes (56). However, all of these images are an unstructured data type, and while computers can readily manage the raw image data, they cannot easily recognize the type of image, annotations on the image, or draw conclusions from the data (56). Thus, the challenges in imaging informatics are to acquire imaging data with high fidelity that accurately represent the image and to process this unstructured data into interpretable content.

## Clinical Informatics

Clinical informatics focuses on data gathered from individual patients (Figure 1B). Clinical informatics data includes any observation of a patient and is defined by the patient in question, the parameter being observed (e.g., liver size, urine glucose value, clinical history, etc.), the qualitative or quantitative value of the parameter being measured (e.g., blood glucose 98 mg/dl), the time when the parameter was observed, and how the parameter was measured. Clinical informatics involves using patient data to make predictions that can help clinicians make more accurate and rapid decisions about diagnosis, treatment, and prognosis of their patients (11). This facilitates clinicians’ use of evidence-based medicine, which allows for data-driven decision making rather than making clinical decisions solely based on general information, personal experience (i.e., what has worked before), or anecdotal evidence (i.e., what experts have found to work in the past).

While some pieces of clinical informatics data are structured (e.g., clinical laboratory findings), much of the data is unstructured, in the form of narrative clinician notes and diagnostic interpretations (e.g., radiology and pathology reports) contained in EHR. These text-based data are difficult to process quickly and reliably because of the lack of standardized reporting protocols across individuals and clinics/hospitals. Manual annotation of clinical records is extremely labor intensive (57). Yet, clinical notes are one of the richest sources of detailed information on disease status and response to treatment for individual patients (58). Recent advances in automated text mining can make the narrative portion of the clinical record computationally accessible, allowing for deeper insights into disease phenotypes. Mining of EHRs requires specific algorithms that use natural language processing (NLP), a group of methods that involve the processing of unstructured text to represent the relevant information in the text with high validity and reliability. While NLP unlocks a wealth of potential clinical data, uncertainty, redundancy, and inefficiency are still major hurdles to the use of this type of data (58). However, the information contained in EHRs is relatively inexpensive to obtain and typically represents more information than can be collected in research studies (59).

## Population Informatics

Epidemiology involves the study of disease prevalence and incidence, as well as the identification of disease risk factors. While all of epidemiology is concerned with identifying and tracking the causes of disease in populations, epidemiologic investigations fall broadly in to three areas: infectious disease epidemiology, genetic epidemiology, and environmental epidemiology and toxicology (Figure 1A). The paradigm of traditional epidemiology—the identification of one to several risk factors associated with disease—has serious limitations, often failing to fully encompass the all risk factors for a disease and how these risk factors are related to one another (60). However, advances in high-throughput technologies and the rapidly growing ‘omics fields now allow for a marked expansion in both the breadth and depth of analysis of health and disease at the population level (61).

### Infectious Disease Epidemiology

A primary emphasis of population health informatics is the study of infectious disease transmission across populations/species, including outbreak investigations and pathogen discovery. These investigations typically begin with spatial analysis, or visualization of the patterns of disease spread, by gathering data at the patient level and integrating this information with data from external sources such as a geographical information system (GIS). The resulting analysis forms charts and/or maps that represent the spread of the disease under study. An example of this type of analysis is https://healthmap.org, which uses informal online sources to identify and monitor disease outbreaks, providing real-time surveillance of emerging public health threats. Following exploration and visualization of spatial data, statistical methods to test the likelihood that an observed spatial or spatio-temporal pattern is a result of chance variation (i.e., spatial scan statistic, space-time scan statistic, and temporal scan statistic) are applied to establish whether a disease is randomly distributed over space, over time, or over space and time (60, 62, 63). Finally, all these data can be used to create predictive models using machine learning or Bayesian algorithms to answer important questions regarding the likely points of control, to predict future directions of spread, and to predict outcomes of different interventions (64).

Increasingly, infectious disease epidemiologic studies are also incorporating sequencing of microbial genomes to follow transmission using traceable differences in pathogen genomes, which provides a high-resolution understanding of transmission and pathogen adaptation and evolution (62) (Figure 1A). For example, Brunker et al., using dog rabies as a model, employed molecular techniques, spatial analyses, and epidemiological models to generate a real-world understanding of infectious disease dynamics, answering key questions about viral spread and persistence (65). NGS can also be used to identify novel pathogens or co-infection of pathogens that result in a synergistic effect or worsening clinical outcomes for affected individuals (66). These methods amplify all the nucleic acid in the sample, including host, viral, and bacterial DNA and RNA, allowing for the identification of novel microbes in the sample. However, identification of a novel microbe does not necessarily indicate pathogenicity. Evidence of pathogenicity has been facilitated by the development of RNAscope^®^ technology for *in situ* hybridization, which allows identification of the novel microbes within affected tissues. The RNAscope^®^ technology utilizes a series of targets for the nucleic acid of the putative pathogen of interest, which is amplified and visualized under a microscope within individual cells (67). This technology can also identify co-infections and suggest the plausible causative agent by quantifying the number of each microbe in a tissue section. RNAscope^®^ has recently been employed as a rapid diagnostic tool during a vesicular disease outbreak in swine to identify a pathogenic virus for which no commercial antibody was available (68).

### Genetic Epidemiology

As the name implies, the primary goal of genetic epidemiology is to identify specific genotypes that increase risk for development of disease (Figure 1A) (69). Genetic epidemiology uses large population data sets to (1) quantify the genetic contribution to disease (*heritability*); (2) identify variability in genetic disease prevalence within and across populations/families (*aggregation*); (3) determine the pattern of inheritance (*segregation*); (4) identify the specific genes and alleles contributing to genetic disease; (5) determine the frequency of genetic disease alleles within and across populations; and (6) predict outcomes for complex genetic diseases based on genetic risk models that incorporate other contaminant risk factors including environmental exposures. Many of these complex genetic risk models also attempt to predict gene-by-environment interactions, and the role of these interactions in disease. With recent advances in genotyping technologies, it has become possible to ascertain large numbers of genotypes (e.g., SNPs) from an individual, and to use these genotypes to identify regions of the genome harboring genetic risk alleles for clinical disease phenotypes using genome wide association (GWAS). GWAS has also been used to identify genomic regions associated with other molecular phenotypes including metabolite abundance (metabolite-GWAS, metabolite quantitative trait loci), gene expression [expression GWAS, expression quantitative trait loci, protein abundance (protein-GWAS), and protein quantitative trait loci] (Figure 1D) (5).

The use of WGS within and across populations enables highly efficient allele discovery and elucidation of the nature of all genetic variation within a population or species. Genetic variation is a key contributor to health and disease, and assessing the “genetic burden” imposed by harmful alleles within the genome has been a major aim of medical genetics for decades. Both whole-genome and whole-exome sequencing are also being used to rapidly identify genetic mutations responsible for rare Mendelian genetic diseases. When accompanied by a comprehensive catalog of common and/or neutral variation from normal healthy individuals within a population, WGS or whole exome sequencing from one to several patients with a simple/monogenic disease can often identify the disease-causing mutations (70–74). From 2009 to 2012, these unbiased mutation discovery approaches were used in human patients to identify >180 novel disease-causing mutations (71). These same approaches can be applied to domestic animal species, in which there are numerous examples of Mendelian traits with high prevalence. As part of the 1,000 Bulls project, WGS from 243 animals from 3 breeds was used to identify rare mutations for curly coat, embryonic death, lethal chondrodysplasia (75), and junctional epidermolysis bullosa (76, 77). Additionally, this data set was used to improve genotype imputation, catalog variants within genomic regions of interest, and quantify inbreeding within cattle populations (78). As similar data are generated in other species, identification of additional mutations influencing disease and other traits of economic importance may be expected.

### Environmental Epidemiology and Toxicology

Environmental epidemiology and toxicology focuses on the discovery of environmental exposures that contribute to disease and the quantification of these exposures, including differences due to geographical location and lifestyle (or management) choices. Environmental exposures can be proximate (e.g., directly leading to a health condition), such as certain chemicals, physical agents, microbiological pathogens, diet, and exercise, or distant, such as climate change or other broad-scale environmental changes (79). Environmental epidemiologists use bio-monitoring (the measurement of the burden of toxic compounds in a biologic sample) to estimate environmental exposures and establish health risks. For example, there is increasing evidence that byproducts of chemical manufacturing, namely persistent organic pollutants, accumulate in human and animal tissue, and act as endocrine disrupting chemicals, interfering with early embryonic development, reproductive development, sexual maturity, and metabolic function (80–82).

The sum of an individual’s total environmental exposures over a lifetime is referred to as the *exposome*. The exposome was first proposed by Wild in 2005 and has been referred to as the nurture part of “nature vs. nurture” (79, 83, 84). The exposome places exposures within the broader context of diet, behavior, and other exogenous and endogenous agents and is divided into three broad categories: the general external environment (shared infectious disease exposure, air pollution, weather, climate change), the specific external environment (infectious agents, chemical contaminants in food or water, approximate environment, occupation, medication exposures), and the internal environment (physical activity, metabolism, inflammation, oxidative stress, body morphology, aging, endogenous hormones, and microflora) (i.e., microbiome, virome, fungome) (83).

## Bringing “Big Data” Together to Understand Health and Disease

The explosion of big data has led to a growing interest in integration of information across the molecular, tissue, patient, and population levels of biomedical informatics data. This assimilation of data is the focus of translational bioinformatics (11). Translational bioinformatics can be defined as the development of analytic and predictive methods and models that optimize the translation of huge volumes of biomedical data into clinical practice (85). The main goal of translational bioinformatics is to answer questions at the clinical level by bridging the gap between disparate data types (11). Translational bioinformatics attempts to address a common set of challenges shared by human and veterinary medicine including (1) improving precision and accuracy of disease diagnosis (particularly early diagnosis through the identification of disease *biomarkers*); (2) choosing the most effective treatments; (3) correctly predicting disease progression and prognosis; (4) understanding disease etiology; and ultimately, (5) preventing disease in subsequent individuals. This aligns with the concept of *precision medicine*, which seeks to individualize medical practice through *deep phenotyping* and *disease sub-classification* to allow for optimal treatment based on an individual’s unique combination of genes, environment, and comorbidities (19). Beyond translational bioinformatics, *network* or *systems medicine* looks to interpret physiologic and pathophysiologic changes within the context of networks using known or predicted relationships between molecules, individuals, and/or populations.

### Deep Phenotyping

Phenotypes are the observable traits of an organism. Phenotypes that result in deviation from normal morphology, physiology, or behavior are the focus of biomedical informatics (59). Disease phenotypes measured at the clinical level are the traditional point of reference and can answer simple questions about the manifestation and severity of clinical disease(s) within an individual, and/or the proportion of individuals within a population displaying or developing disease (prevalence and incidence). However, the advent of high-throughput technologies, which allow for the collection of a large number of phenotypes from patients at the clinical, tissue, and molecular levels, has led to the concept of “deep phenotyping” (Figures 1B–D) (86). Deep phenotyping provides a more complete clinical picture of an individual patient using the collection of high-resolution phenotypes relevant to a clinical syndrome or disease and results in a level of phenotypic detail that was not previously possible (87). Deep phenotyping emphasizes quantitative phenotypic measures because they better differentiate between marginal and severe cases, resulting in more powerful statistical comparisons than qualitative measures (i.e., yes/no, clinical grading scales, etc.) (87). Deep phenotyping of an individual at any given point in time allows for a comprehensive and thorough description of the individual’s physical state; a complete description of an individual’s phenotype has been described as the *phenome* (88).

Deep phenotyping across a group of patients allows for a more thorough disease definition and an understanding of the full spectrum of abnormalities for a given condition (59, 86). Ideally, deep phenotyping would be repeated over time, providing a longitudinal understanding of disease progression/pathophysiology and permitting for early biomarker discovery (61). Deep phenotyping also allows for a clearer separation of different diseases/syndromes that superficially appear to have similar (or even identical) clinical presentations.

### Disease Subclassification

**Figure 3:**
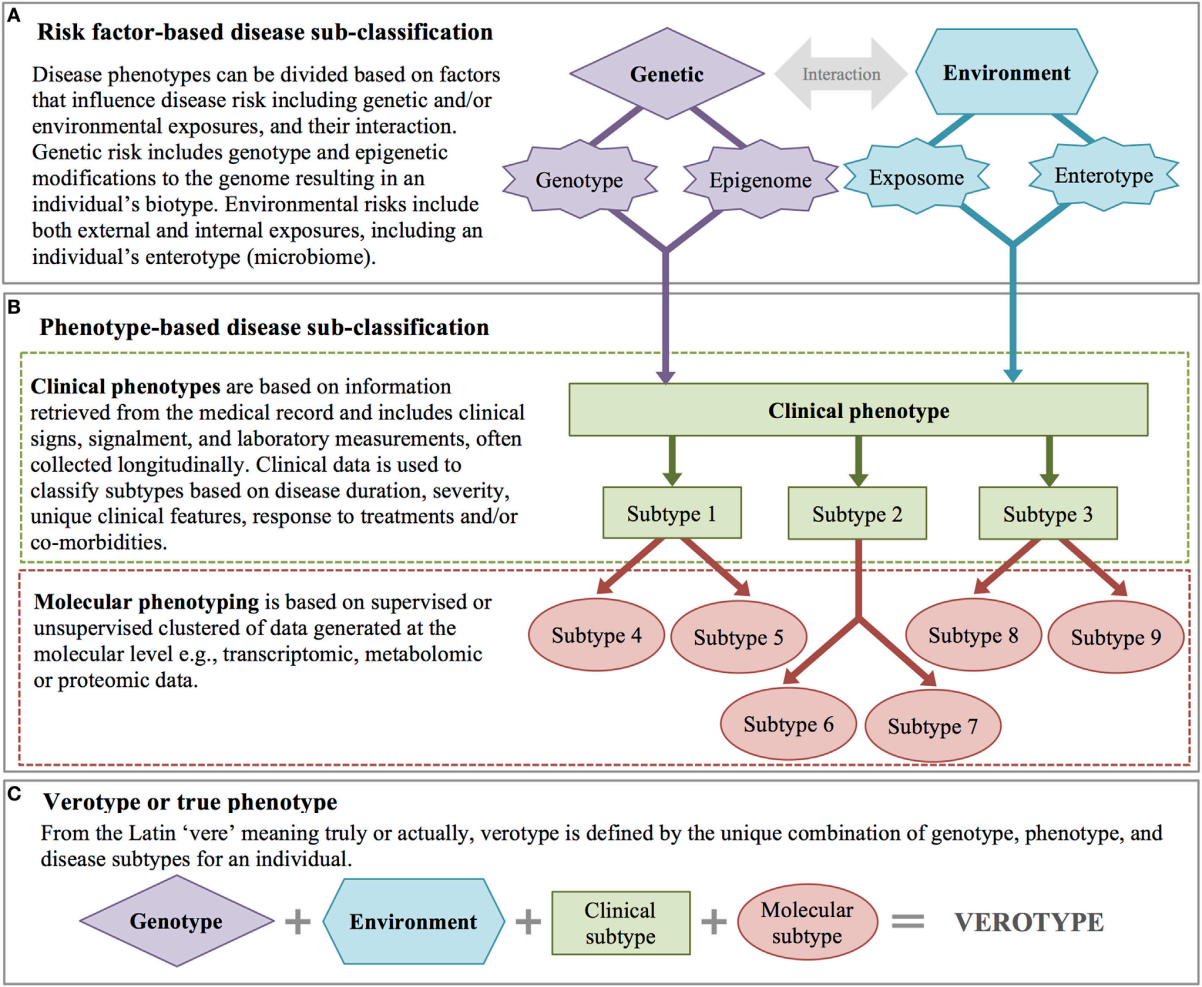
Disease subclassification. **(A)** Risk factor-based disease subclassification includes factors that increase disease risk (even prior to disease). **(B)** Phenotype-based disease subclassification includes both clinical factors and molecular phenotypes. **(C)** A patient’s verotype or true phenotype is the result of risk factors and molecular and clinical subtypes.

Phenotype and disease subclassification are a fundamental challenge in precision medicine. If a heterogeneous group of patients can be separated in to appropriate subtypes, more effective individualized treatment becomes possible. Subclassification is also a powerful tool for translational research, as classifying patients based on differences in deep phenotypes can lead to better patient selection for clinical trials and inclusion in other research studies such as GWAS. Diseases are subclassified *a priori* either by the presence or absence of particular risk factors (Figure 3A) or based on clinical and/or molecular phenotypes (Figure 3B).

*A priori* classification takes into account genetic and environmental risk factors. Genetic risk factors include known genetic risk alleles with a disease-related phenotype (regardless of disease state), genetic background (e.g., ethnicity in humans, breed in domestic animals), and/or known epigenetic modifications that affect phenotypic expression (89). An individual’s biotype represents the sum of that individual’s genetic potential for disease. Environmental risk factors may include exposure to particular endocrine disrupting chemical(s), other pollutants, or infectious disease agent(s), as well as a patient’s enterotype (based on their gut microbiome and defined by the abundance of certain bacterial genus and species) (90).

With the widespread adoption of the EHR, methods to extract qualitative and quantitative data for disease subtyping have become an active area of research (91). High-throughput clinical phenotyping algorithms combine information from structured data (e.g., laboratory values) and unstructured data (e.g., clinical signs, signalment, results of imaging studies, response to specific interventions) to annotate clinical features from the EHR. Raw EHR data are then characterized by calculating the frequencies of clinical features, and associations between features such as disease co-occurrences are identified. These data can also be used to predict outcomes (i.e., response to treatment, prognosis, adverse drug reactions, etc.). Longitudinal data can be used to calculate disease duration and catalog progression. Patients can be classified using supervised or unsupervised clustering machine learning methods (91), and clinical subtypes are created based on factors such as disease duration (acute vs. chronic), severity, and the presence and absence of particular clinical signs or disease comorbidities.

With the advent of high-throughput molecular measures, there has been an increased interest in further subclassification of disease based on molecular phenotypes, including genomic, transcriptomic, metabolomic, and proteomic data. Similar to clinical features, disease subclassification based on molecular phenotypes is achieved either through supervised (based on clinical hypotheses) or unsupervised clustering methods (60). Ultimately, disease phenotypes are subclassified based on integrative analysis across both clinical and molecular features. Integration of these heterogeneous data (continuous and categorical measures) and the combination of data that are measured at a single point in time (e.g., gender, DNA sequence) with those measured longitudinally present unique computational challenges. However, capitalizing on both types of data advances human and veterinary medicine toward the ultimate goal of understanding and classifying diseases based on their specific pathophysiology, a subclassification scheme referred to as an *endotype* (92). While endotype may be considered the disease-centric definition of the “ultimate” phenotype, the patient-centric definition is the *verotype*. The verotype is the unique combination of genotype, phenotype, and disease subtypes within an individual, in other words, the entire sum of a patient’s risk factors and clinical and molecular phenotypes (Figure 3C).

### Network (Systems) Medicine

**Figure 4:**
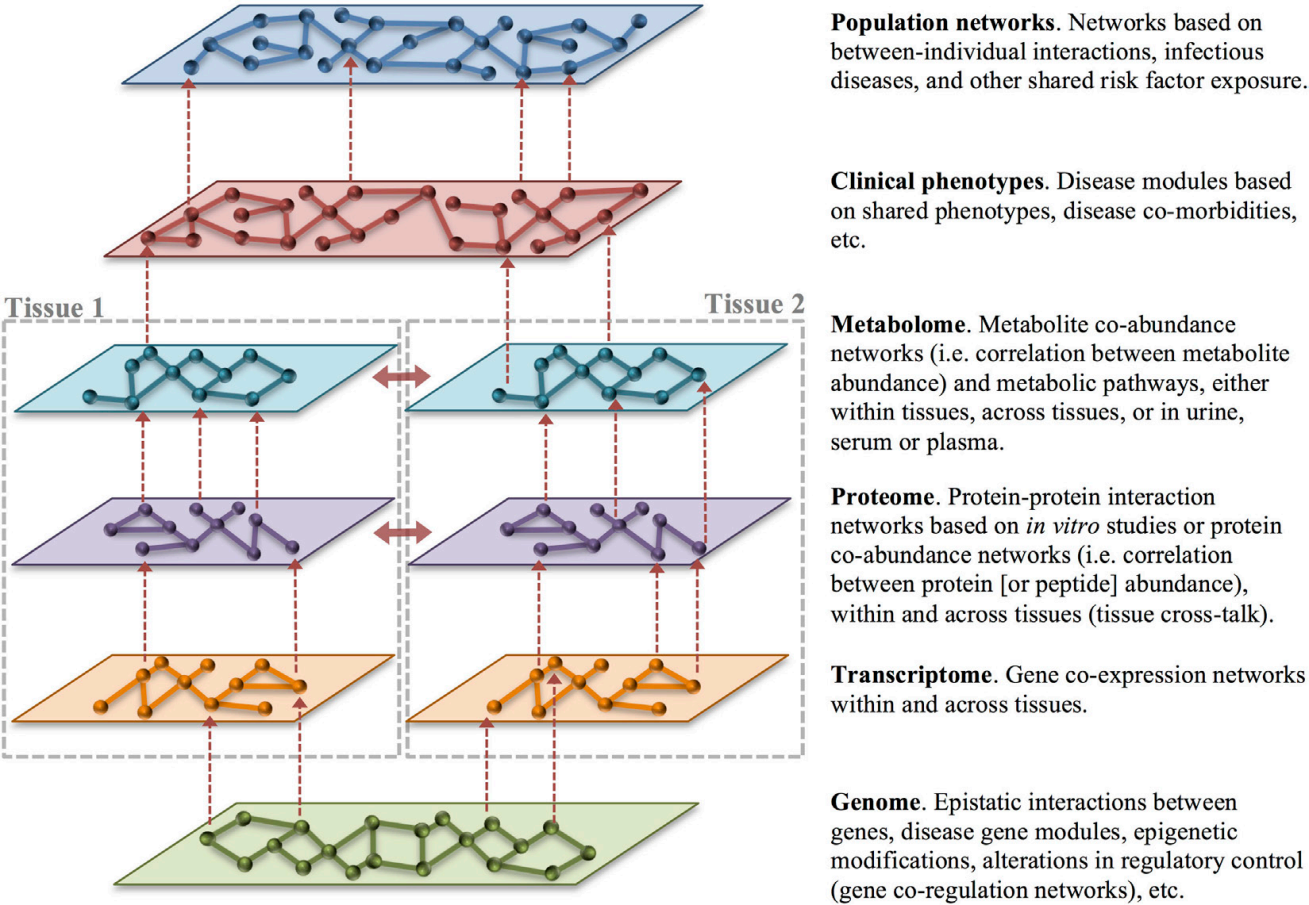
Multilayer disease modules. Organizing heterogeneous big data into biologic networks can lead to a deeper understanding of normal function and dysfunction in disease. Networks consist of nodes representing an object of interest that are connected by edges that capture the relationship between the nodes. Networks can be built with data gathered across layers and tissues as well as across individuals. Vertical integration of networks across the levels of health-care big data (dashed red arrows) is an important goal of translational bioinformatics. Integration of data across tissues (solid red arrows) also provides an opportunity to understand tissue cross-talk in health and disease. In particular, changes in cross-talking proteins may signal rewiring in disease.

**Figure 5:**
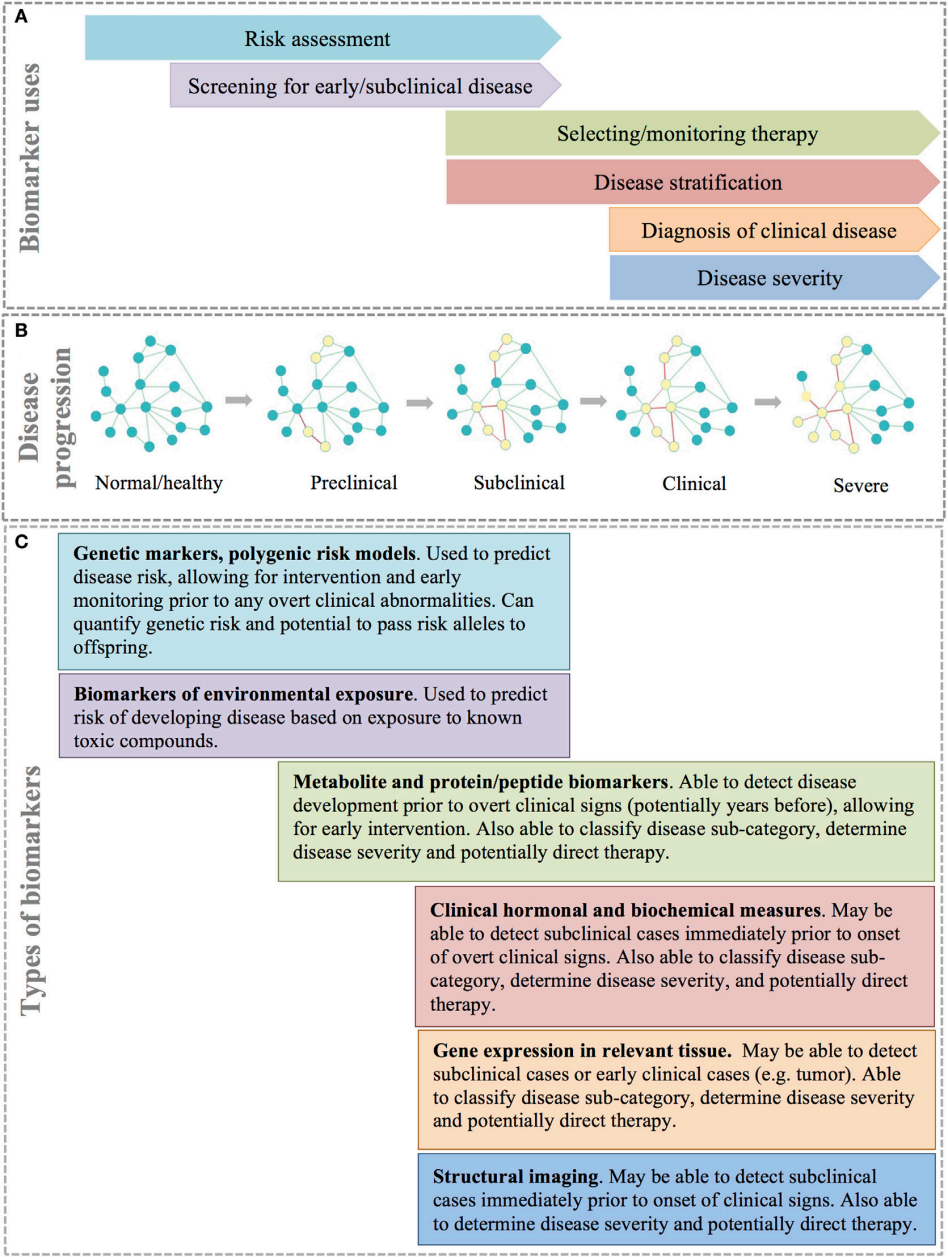
Types and uses of disease biomarkers. **(A)** Different biomarkers are used for different reasons at different points in disease progression. **(B)** Disease progression can be thought of as a continuous process that is the result of rewiring of important molecular, cellular, or tissue networks, which results in progression from a healthy state to severe clinical disease. **(C)** A combination of biomarker types allows for identification of at risk or disease patients at different stages of disease.

A natural extension of disease subclassification and deep phenotyping is organizing these data into biologic networks. A network is simply the graphical representation of the relationships between objects, where the nodes represent the objects of interest (cell, molecule, individual), and the edges represent the interactions between them (mathematical correlations, physical contact, etc.). All organisms consist of a multitude of interconnected biological networks including networks within and between cells and within and between tissues. Network (systems) medicine is a rapidly growing discipline based on combining high-throughput molecular data with clinical and functional studies (93). A central tenet of network medicine (and more globally, systems biology) is that to understand biologic systems they must be studied within the framework of molecular, cellular, and tissue interconnectivity (93). Network medicine capitalizes on data from *in vivo, ex vivo*, and *in vitro* experiments as well as *in silico* analyses to create biological networks (94). Biological networks are graphical representations of the interactions between molecular or other disease components, organizing this data into a template that allows for a better understanding of how these variables interact in health and disease (95). This is particularly important for complex diseases, which cannot be fully explained by focusing on single genes, molecules, or environmental risk factors (collectively, disease variables), but rather by examining all of these components and the network interactions that arise between them (Figure 4) (96, 97). Complex diseases that are the result of many risk factors often have insidious onset and unpredictable progression because they are caused by perturbations of complex intracellular and intercellular networks that link the cellular, tissue, and organ components of the system (98). Progression from normal to disease state is a dynamic process in which normal molecular networks are progressively disrupted until a tipping-point is reached (17, 99, 100), resulting in the breakdown in functional modules (or sub-networks) connecting cellular or organ components (Figure 5B). Understanding these functionally relevant subnetworks and how they break down (or re-wire) over time is key to identifying early disease processes and potential targets for intervention, including drug targets (17, 99). Thinking of progression in terms of networks is most understandable for diseases caused by the combined actions of multiple genes and environmental factors; however, it is equally important to understand networks in healthy individuals so that disease perturbations can be recognized. In humans, even social networks (human-to-human interactions) have been shown to be extremely important in disease risk, not only in the spread of pathogens but also in the occurrence of non-infectious diseases such as obesity (99).

Disease networks can be created from molecular data, such as genomic, transcriptomic, proteomic or metabolomic data, from deep phenotyping or other clinical data, and from population epidemiologic data—or any combination thereof. Molecular, cellular, tissue, and interindividual networks provide context-specific insight into the mechanism of disease. For example, to better understand the relationship between genotype and phenotype in complex genetic disease, one approach is to examine the relationships among genes or intermediate phenotypes across different types of ‘omics data, including gene co-expression networks constructed from the correlation of gene expression across samples and/or tissues. Tissue gene co-expression networks can provide a basis for the prioritization of candidate genes for disease risk by capitalizing on the biologic connections between known disease genes and the remaining genes that occur within a network. If a particular gene is known to affect the phenotype of interest or a related phenotype, this can point to neighboring genes within the network as likely candidates to be involved in an important biologic pathway using a guilt-by-association principle (101). Further, because genes that are strongly co-regulated often occur within the same biologic pathway, co-regulation networks can often give context to genes with unknown function (96).

Protein–protein interaction networks are mathematical models of the physical contacts between proteins. These protein–protein interactions are critical to almost every cellular process, and similar to gene co-expression networks, understanding these interactions can clarify the function of proteins and identify important cellular processes in health and disease (94). Similarly, metabolite co-abundance networks constructed from the correlation of metabolite abundance across samples provide contextual information and identify biologic pathways. Layering different forms of functional information, such as metabolite or protein co-abundance networks, over gene co-expression networks can help compensate for missing or unreliable information from gene expression data. Most importantly, multiple sources of evidence pointing to the same gene or pathway increases confidence in their role in the clinical phenotype of interest. Building multilayer disease modules across molecular, clinical, and population data is the ultimate goal of translational bioinformatics and network medicine (Figure 4).

In additional to patient-centered molecular and clinical data, networks have also been created at the level of disease co-morbidity (99). Genes associated with the development of the same disease or phenotypically similar diseases often co-localize within a protein–protein interaction network (99). This information can then be used to develop a disease model. Other genes within the network can then be assumed to be potentially important for phenotypically related diseases.

Networks in infectious disease epidemiology are another extension of network medicine. In infectious disease transmission networks, the nodes represent individuals or groups of individuals, and the edges represent either contact between the individuals (*contact network*) or known or hypothesized disease transmission events (*transmission network*) (102). *Contact networks* are used to model the transmission rate of infection through a population, based on the underlying assumption that increasing the number of contacts between individuals will increase the transmission rate. Factors influencing contact such as animal behavior and animal movements are modeled to understand contact rate and the likelihood of contact between individuals. *Transmission networks* are a subset of contact networks—factors affecting the likelihood that an individual is exposed, becomes infected and subsequently transmits a pathogen can be modeled and adjusted for a myriad of factors such as age, sex, host genotype, and immunocompetency (e.g., resistance through prior infection, vaccination, or immnocompromise due to stress, pregnancy, contaminant infection, etc.) (102). As noted above, transmission networks can also be reconstructed using pathogen genetic markers or sequencing of pathogen nucleic acids (DNA or RNA) to reconstruct transmission events by constructing phylogenic trees of the relationships between the pathogens obtained from individual hosts or host populations (64, 102). Both contact and transmission networks can be used to identify places within the network that pose the greatest risk for spread of the pathogen. By modeling the impact of interventional measures such as vaccination, removal of individuals form the population, or limiting potential contact, specific, timely recommendations can be made to limit disease transmission (102).

### Biomarkers

Disease subtyping, deep phenotyping, and network medicine each aim to better describe disease and improve understanding of disease etiology, leading to superior therapeutics and the potential for early disease intervention. However, translation of these ideas to the clinical patient requires sensitive, specific, and relatively inexpensive diagnostic tests that can detect preclinical, subclinical, or clinical disease and accurately classify patients. A biomarker is any substance or process that can be measured in a biological specimen that is reliability correlated to a patient’s disease state and/or clinical response (103). Biomarkers are commonly important molecules, such as DNA, RNA, metabolites, or proteins/peptides that are found circulating in blood or within tissues. However, biomarkers can also be based on other diagnostic modalities such as structural imaging (Figure 5) (104). Ideally, the assay of biomarkers should be minimally invasive (i.e., measurable in peripheral blood or urine); however, for many disease processes, such as cancer, biomarkers based on biopsy of solid tissues are often more specific and informative. Biomarkers are a key component of precision medicine and are used for several purposes including risk assessment, screening for preclinical or subclinical disease, diagnosis of clinical disease, disease stratification, selecting and/or monitoring response to therapy, and predicting disease progression and prognosis (104, 105).

Risk assessment with biomarkers is performed prior to disease onset with the goal of stratifying individuals into groups to identify those individuals most likely to benefit from early intervention, prevention strategies, or additional diagnostic screening (105). For example, women with mutations in the *BRCA1* or *BRCA2* genes have a 45–65% risk of developing breast cancer during their lifetime (106, 107). Guidelines for women positive for mutations in either of these genes include mammography at an increased frequency starting at an earlier age in an effort to diagnose and treat the disease early in the clinical course (107).

Biomarkers are also used as screening tests for preclinical and subclinical disease, or even early stage clinical disease, while the patient is typically asymptomatic (Figure 5A). Biomarkers used for screening should be highly sensitive and have reasonable specificity and predictive values to be useful in clinical practice. Further, the benefits of early intervention (including better disease outcomes) should outweigh the costs (and risks) of performing the screening test (105).

After the onset of clinical signs, diagnostic biomarkers are used to definitively identify the cause of disease (Figures 5A,B).They are also often used to determine the severity of the disease based on quantitative measures; for example, the magnitude of elevation in circulating bile acids is correlated to the severity of liver disease. Over time, biomarkers can be used to determine disease progression and, ultimately, predict prognosis in a defined clinical population (105).

In addition to quantifying disease severity and progression, biomarkers in human medicine are increasingly important in subclassifying patients, particularly into groups that are likely to respond to treatment. The traditional approach to drug selection for most diseases is empirical, with treatment continued or changed until a satisfactory clinical response is attained (103). With patient subtyping and improved understanding of the mechanisms underlying diseases, there is an opportunity to identify targeted therapies that will be both safe and efficacious in individual patients. An area in which this type of targeting has been particularly pursued is in the choice of a particular chemotherapy regimen based on molecular markers identified in a patient’s tumor (104). While this approach is still in its infancy in veterinary medicine, there are examples of known population- and patient-specific efficacy and toxicity, such as variable absorption and conversion of prednisone to prednisolone in horses (108), or ivermectin toxicity in collies with a loss of function mutation in the *MDR1* gene (109).

While the idea of one or more simple biomarkers that can accurately screen, diagnose, monitor, and predict prognosis of disease is appealing, the reality is that for diagnostic biomarkers to be successfully utilized for complex diseases, they must be multifaceted (Figure 5C). The earliest prediction of increased disease risk can be achieved using genetic testing. An individual’s genetic risk for disease is defined by the individual’s multi-locus genotype (110). Since baseline genetic risk remains unchanged throughout an individual’s lifetime, it can be predicted at birth prior to environmental exposure (110). Polygenic risk models can easily be built by summing the number of risk alleles across loci, or by summing genotypic likelihood ratios (for binary traits) or genotypic effect estimates (for quantitative traits) across loci (111, 112). Polygenic risk models are able to predict risk prior to any detectable signs of disease and can identify genetic alleles that can be passed to offspring. However, polygenic risk models are not capable of identifying disease progression to subclinical or clinical disease. Further, polygenic risk models may or may not be useful in disease subclassification or for identifying targeted therapeutic regimens or monitoring response to therapy. However, other biomarkers such as gene expression, metabolites, proteins, and clinical measurements (including imaging) have the potential to overcome the diagnostic limitations of polygenic risk models (Figure 5) (113). These biomarkers may be detectable during the subclinical or early clinical phases of a disease and can help to subclassify disease, determine disease severity, and perhaps guide therapeutic decision-making.

**Figure 6:**
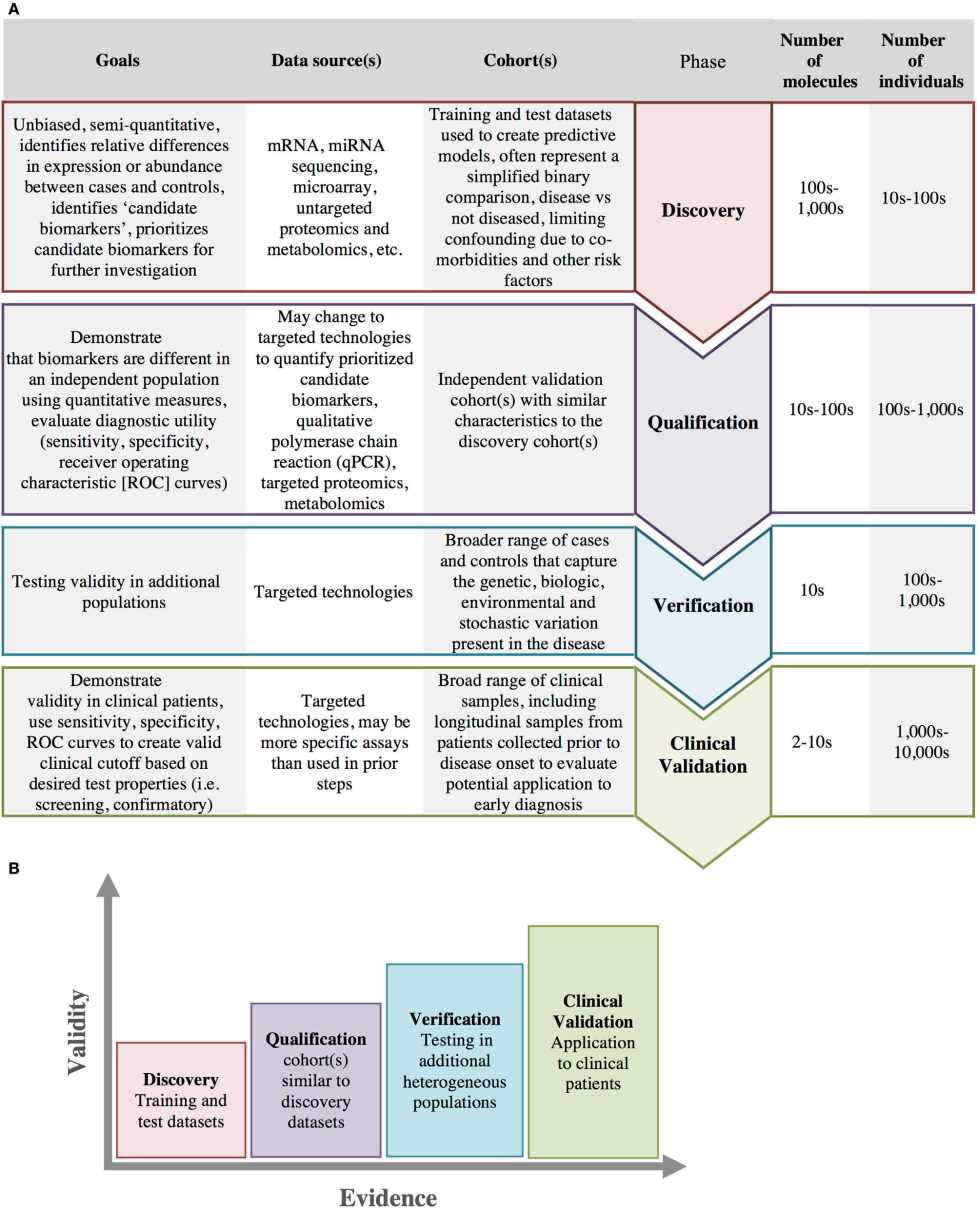
Biomarker development. **(A)** Biomarker development progresses through several stages from initial discovery to clinical validation with increasing number of individuals and a decreasing number of biomarkers at each stage in the process. **(B)** As biomarkers progress through each of these stages, there is an increasing amount of evidence and increasing clinical validity in support of the biomarkers use.

Ideally, diagnostic methods would include a profile of several types of biomarkers able to identify patients across the spectrum of disease progression (114). The steps necessary for the identification and validation of biomarkers are depicted in Figure 6A and include discovery, verification, qualification, and clinical validation. With each progressive step more evidence is accumulated and the validity of the biomarker for clinical use is increased (Figure 6B). While biomarker studies typically leverage molecular data to identify at-risk individuals, clinical/medical decision-making can also be supported through the integration of clinical data into clinical decision support systems (CDSS).

### Clinical Decision Support Systems

Medical decision-making is complex, requiring a vast amount of knowledge to solve even what appear to be simple problems (115). While big data allows rapid accumulation of data from hundreds to thousands of individual patients, the identification of patient clusters, and the development of more accurate predictive models (20), the increased complexity of disease classification and potential therapies have the potential to surpass the clinician’s ability to multitask and apply evidence-based clinical reasoning (116). CDSS attempt to overcome these limitations by providing computational algorithms that assist clinicians to apply the vast amount of knowledge being generated by big data methods (115). CDSSs use a variety of computational techniques such as rule-based systems, heuristics, fuzzy logic, artificial neural networks, and Bayesian networks to make recommendations regarding disease screening, appropriate diagnostic tests, and disease etiology, as well as to predict outcomes, recommend treatments, and prevent clinical errors, thus improving patient care and safety (117).

## Data-Driven Science: A Paradigm Shift

**Figure 7:**
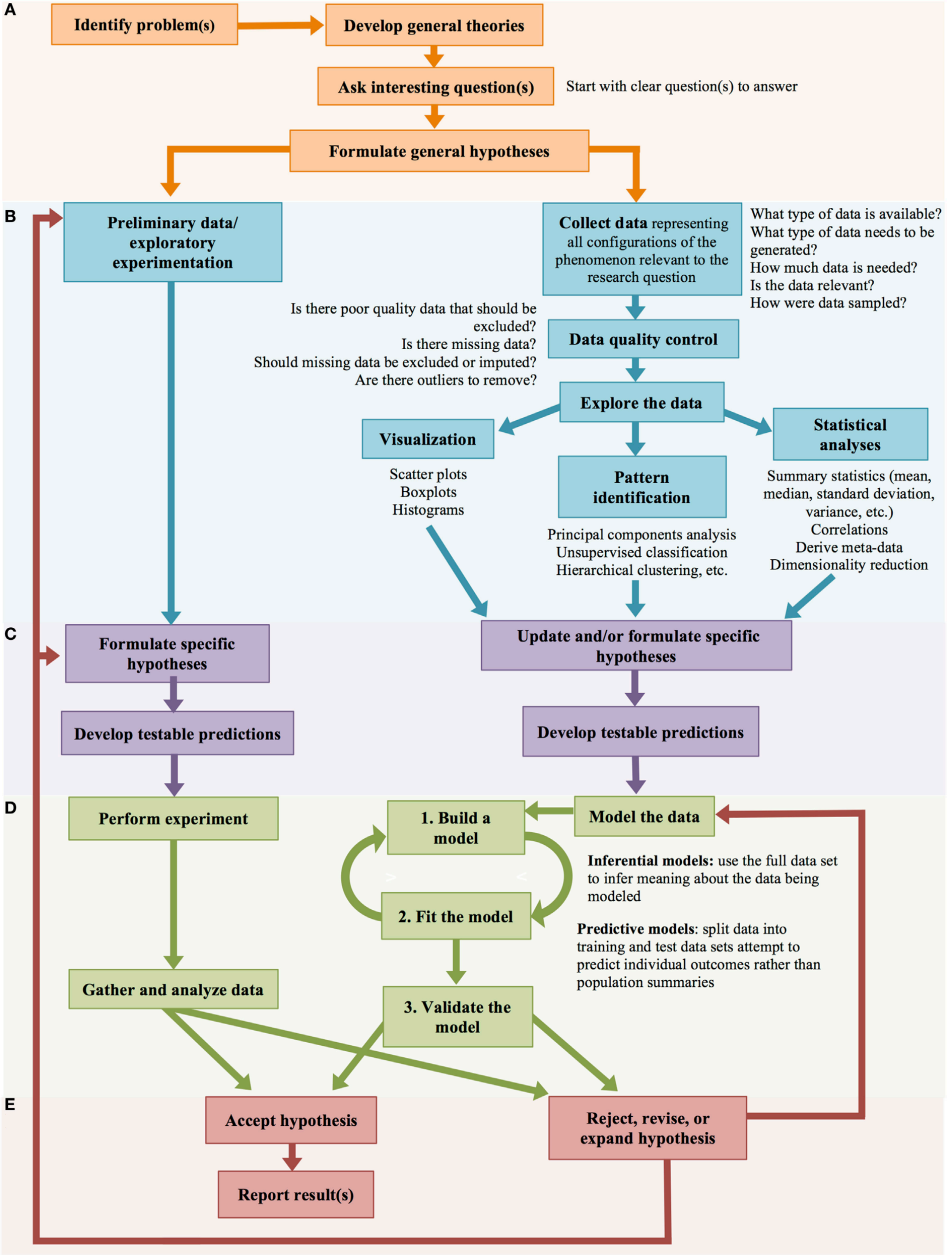
Big data scientific method. Hypothesis-driven and data-driven scientific methods progress through parallel stages. **(A)** Framing the problem and general hypotheses. **(B)**. Data collection and exploratory experimentation/analysis. **(C)** Formulation of specific hypotheses. **(D)** Testing the hypotheses. **(E)** Accepting or rejecting the hypotheses.

With the increasing capabilities of computers for data storage and processing, biology has increasingly become a data-intensive science. Data-intensive science represents a paradigm shift for biology—it asks different kinds of questions and performs science with a different (albeit partially parallel) process compared to theory- or hypothesis-driven science (Figure 7).

Big data-driven science is quantitative and often unbiased by prior knowledge—the data speaks for itself. Data-driven biology looks at the system as a whole; these approaches acknowledge that biology does not occur in an experimentally controlled system, but is messy and complicated. Contrary to what many hypothesis-driven scientists argue; data-driven research is not entirely hypothesis-free. Data-driven approaches often start with a broad hypothesis and collect or generate large volumes of data relevant to that hypothesis. These data are then explored to generate more specific and mechanistic hypotheses through eliminative induction or abductive reasoning (Figure 7) (21). Data-driven science removes the assumption that the scientist has adequate biologic insight to generate the best specific, testable hypotheses; instead, it assumes that the scientist’s understanding of complex biologic systems is rudimentary and, therefore, uses data to generate specific hypotheses (21).

Both data-driven and hypothesis-driven research start by identifying an interesting research problem and the intellectual process of developing general theories regarding the problem based on prior knowledge and previous research efforts. In both instances, the researcher works to identify scientific questions that need to be answered and formulates general hypotheses regarding the research problem (Figure 7A) (21). Hypothesis-driven and data-driven science diverge in the next step (Figure 7B), where hypothesis-driven scientists perform exploratory experimentation (through laboratory experiments, or mental deduction), and then formulate specific hypotheses (Figure 7C).

In contrast, in data-driven science, data are generated (through experimentation) or collected (observational data), which represents all possible configurations of data that may be relevant to the research problem (21). To satisfy these requirements, the data are typically high-dimensional (involving many parameters) and have a large number of observations (118). Data collection is a critical step in which the scientist must be able to ensure that the appropriate amount of data that captures the relevant variables that may affect the outcome(s) of interest are included (118). It is also critical that data are collected in a manner that minimizes sources of bias and potential confounding factors.

A scientist’s decision regarding the relevant data to collect typically stems from a broad hypothesis, for example: “skeletal muscle gene expression profiles will differ between patients with rhabdomyolysis and normal patients,” or “disease outbreaks in swine finishing facilities will be related to animal movement.” These general hypotheses frame the problems or research questions and guide data collection (RNA sequencing in skeletal muscle tissue, or animal movement data), but avoid making more specific causal or testable hypotheses about the relationships between variables in the data (which genes will have altered expression, how animal movement is increasing disease risk). Data-driven science takes several steps to explore the data before arriving at more specific hypotheses regarding the relationships between the variables (119). The data undergoes rigorous quality control, where poor quality and irrelevant data are excluded and missing data are imputed (filled in with an estimated value) if necessary. From here, the data are explored by three general methods. First, the data may be visualized through scatter plots, boxplots, histograms, etc., to begin to conceptualize important trends in the data. Second, patterns in the data are explored through both using supervised methods such as principal components analysis, and unsupervised means such as hierarchal clustering, K means clustering, and self-organizing maps (2). Finally, statistical methods such as summary statistics (means, SDs, etc.), correlations between variables, and correlations between data and metadata are used to begin to understand relationships between the variables, to identify key predictive inputs. In this method, variables are transformed if necessary, and dimensionality reduction techniques may be applied to make modeling more computationally feasible (119) (Figure 7B). After data exploration, the data-scientist develops more specific hypotheses regarding the relationships between the variables, including hypotheses about causal relationships (Figure 7C) (21). In purely observational big data studies, inferential or predictive modeling may not be pursued.

In the next step of the scientific process both hypothesis- and data-driven scientists (in experimental or quasi-experimental studies) test, their specific hypotheses, either by experimentation (hypothesis-driven science) or by computationally modeling the data (data-driven science) (Figure 7D). Data-intensive models are concerned with relating an outcome of interest (*y*) with a large number of input or predictor variables (*x*), to determine the nature of the dependence between the outcome and the predictor(s) (i.e., *y* in relation to *x*) (118); for example, modeling the relationship between selected clinical signs or molecular measures from patients (the predictors, *x*) with likelihood of survival or response to a particular treatment (the outcome, *y*) (120). Data-driven modeling includes either inferential models, which use the use the full data set to infer meaning about the cohort being modeled, or predictive models, which attempt to predict outcomes for individuals rather than providing summaries of the population (or data cohort) (121). Due to the complexity and large numbers of predictor variables, data-driven models are often developed using machine-learning algorithms (e.g., partial least squares discriminate analysis, classification and regression trees, support vector machines, random forests, neural networks, etc.). To develop a predictive model, the model is trained on a portion of the full data set (training data) and then tested (validated) against test data that was withheld from the full data set during model building specifically for the purpose of testing the model (2). Model building and testing is an iterative process in which variables are added or removed from the model until optimal model fit is obtained (variable or “feature” selection) (Figure 7D). Typically, modeling efforts focus on classification or regression problems. Regression problems typically involve on estimating the strength and directionality of the relationship between *x* and *y*, whereas classification involves building models that can assign a new observation *x* (typically a patient) to a known class (e.g., likely has the diagnosis, likely to respond to treatment) (120).

The final steps of the scientific process for hypothesis- and data-driven are again the same—after experimentation and data collection in one case, or data modeling in the other, hypotheses are either accepted or rejected. If a hypothesis is rejected, the data-driven scientist often returns to the same data to repeat modeling to test a new hypothesis, whereas the hypothesis-driven scientist returns to experimentation to test a new or revised hypothesis (Figure 7E).

## Challenges and Opportunities for Big Data in One Medicine

The promise of big data in one medicine is that terabytes to petabytes of data can be used to provide clues for everything from transmission of rabies virus to the genes and alleles responsible for osteochondrosis risk. Big data has the potential to improve the quality, safety, and efficiency of clinical care, thereby enhancing clinical outcomes or improving population health outcomes (122). Big data should be able to capture insights from data gathered from research and clinical patients and combine these data to develop an evidence-based learning model to improve the practice of human and veterinary medicine (122). However, while big data holds many promises for one medicine, there are also many challenges associated with the analysis of real, messy, incomplete, and heterogeneous big data (123).

Data heterogeneity can be a hurdle to meaningful integration of data from different sources, particularly when these data vary in scale or frequency of sampling, or are removed from each other in terms of the biologic processes (2). Datasets in which a large number of variables are measured on a small number of individuals (big *p*/small *n*), a common scenario in ‘omics studies, particularly in veterinary species, are extremely prone to statistical issues including estimate instability, model over-fitting, and large SEs (2). ‘Omics studies such as GWAS and untargeted metabolomics studies are also prone to high rates of false-positives due to chance alone (multiple testing problem), meaning that either very stringent statistical significance cut-off are required, or a high false discovery rate must be tolerated (124).

Big data approaches and the ability to integrate information from diverse sources provides an opportunity to capitalize on the large number of individual, small scale data sets produced by individual groups in comparative medicine. These published “long-tail datasets” are small individually, but collectively represent the majority of biomedical data (125). There is also a large amount of “dark data” in science; that is, those datasets that are not put into the public domain because they failed to support a hypothesis, did not generate a “sufficient” amount of new knowledge, or were otherwise “un-publishable” (125). Sharing of data that is incomplete, poorly described, of low resolution or quality has little value and can lead to inappropriate re-use of data and drawing inaccurate conclusions from the data (126). However, as best practices are developed for data collection, storage, and quality control of new data, these methods can also be applied to consolidation of both “long-tail datasets” and “dark data,” thereby allowing these data to be used. Although individually under-powered, when combined, these data could allow for comparisons to be made between different patient cohorts, or in similar conditions across species (125). There is a particular need to capitalize on these data in veterinary research, where limited funding and limited access to patients with specific, well-defined phenotypes often limits the samples sizes within a particular study. Therefore, making these raw data discoverable, accessible (data and related metadata), intelligible (to humans and computers), and reusable is an opportunity that veterinary medicine cannot afford to ignore (126). Data sharing also has the potential to directly impact evidence-based medicine. With the increasingly widespread adoption of EHR in medicine, establishing mechanisms to share this data across institutions is another vital opportunity. As stated earlier, the information contained in EHRs is relatively inexpensive to obtain and often represents more information than is collected in research studies (59). These data are often more directly related to clinical patients than data collected as a part of research studies which, by their nature, are biased to more homogenous research cohorts and may or may not be representative of the clinical patient in question (12, 127).

Although the potential benefits of big data approaches in human medicine to improve human health are self-evident, the use of similar approaches in veterinary medicine is particularity important if comparative medicine is going to fully capitalize on the promise of big data. The rapid development of minimally invasive quantitative methods to capture biologic big data, in particular, advances that remove the need for species-specific tools (such as NGS), represent a new opportunity for one medicine. A decade ago, domestic animal genomics (and other high-throughput technologies) lagged behind what was feasible in humans and a select few model organisms because of the time and cost associated with the development and optimization of species-specific tools such as SNP genotyping arrays, or validation of ELISAs designed for other species. Now, the gathering of high-throughput data, deep phenotyping, and disease subclassification that are available to researchers in human health are also available to veterinary medicine. This means that one medicine can capitalize on information from naturally occurring spontaneous models of disease in domestic animal species and gather data that equals or exceeds what can be collected in human patients. In addition, factors such as multiple births, short generation time, and plentiful half- and full-siblings, greater control over factors that are difficult to control in human populations (e.g., diet, breeding, etc.), and the opportunity to collect samples that might be deemed too invasive for human patients are all benefits of studying disease in domestic animals (128). While laboratory species have traditionally been looked to as models for studying diseases important to humans, companion animals and livestock have distinct advantages, in that they are longer-lived, better recapitulate athletic and injury phenotypes in humans (e.g., arthritis), and provide an opportunity to study zoonoses and diseases of shared environments including allergens and exposure to environmental hazards (e.g., toxins, radiation, etc.) (128).

Capitalizing on the promise of big data in comparative medicine requires training a generation of “data-clinician-scientists” that are able to harness big data and translate it into clinically applicable information. These researchers must be comfortable with multi-level, multi-modal, large *p*/large *n* data for the investigation of disease processes (126), and will require familiarity with sophisticated computational software solutions, including the ability to write computer code and appropriately apply statistics, in order to extract biological insights from large data sets. Further, integrating discoveries into clinical practice requires that practitioners be able to translate research findings into specific actions. This means not only having accurate diagnostic tests that can identify patients who will benefit from particular interventions but also clinicians that understand and can interpret the sensitivity and specificity of multi-marker and/or multi-model tests. Realistically, research teams with a combination of computational skills and medical expertise will need to come together to translate big data discoveries into clinical practice; thus, big data researchers that come from a computational background must be able to speak intelligently with subject matter experts in human health care and veterinary medicine (2)—and *vice versa*—with the common goal of propelling “one medicine” forward.

## Author Contributions

MM and AM developed the concepts and edited the manuscript. MM wrote the first draft of the manuscript and prepared the figures.

## Conflict of Interest Statement

The authors declare that the research was conducted in the absence of any commercial or financial relationships that could be construed as a potential conflict of interest.
